# Supplementary material for: The effects of genital myiasis on the diversity of the vaginal microbiota in female Bactrian camels
Source: BMC Vet Res. 2022 Mar 5;18:87. doi: 10.1186/s12917-022-03189-5 (PMC8897907; doi:10.1186/s12917-022-03189-5)
Supplement: Supplementary file 5 — Additional file 5. [file 12917_2022_3189_MOESM5_ESM.zip › MPL201709200_16s_yy/Treat1/C07_picrust/KEGG_L2_Cellular_Processes.pdf]

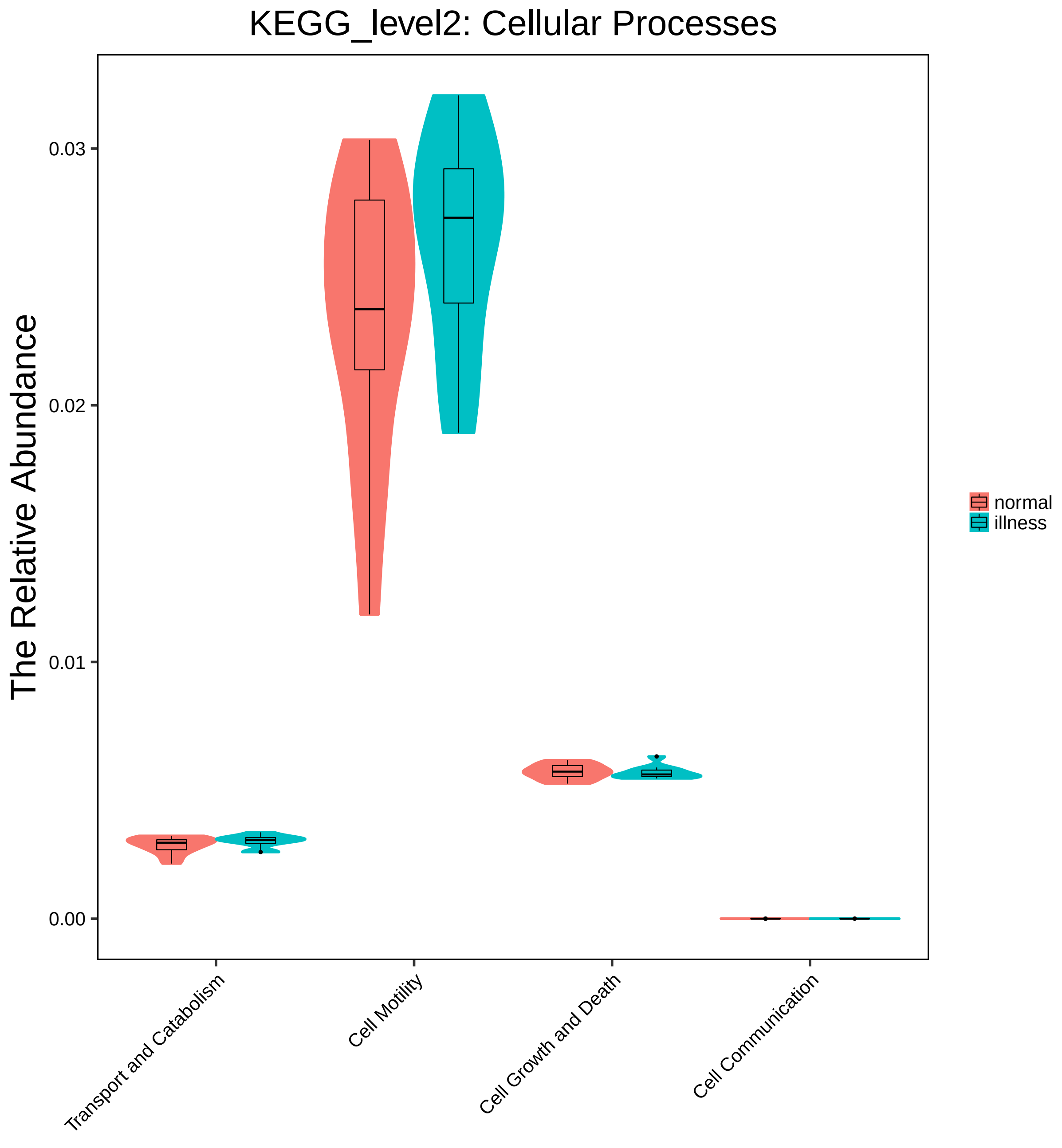
KEGG_level2: Cellular Processes
The Relative Abundance
0.03
0.02
0.01
0.00
Transport and Catabolism
Cell Motility
Cell Growth and Death
Cell Communication
normal
illness
